# Novel Blood-Based Extracellular Vesicle-Derived Biomarkers in Small Cell Lung Cancer Identified via Proximity Extension Assay

**DOI:** 10.3390/cancers18040580

**Published:** 2026-02-10

**Authors:** Hubert Krzyslak, Weronika Maria Szejniuk, Marwan Malluhi, Henrik Steglich-Arnholm, Ursula Falkmer, Jonas Ellegaard Mortensen, Søren Risom Kristensen, Shona Pedersen

**Affiliations:** 1 Department of Clinical Biochemistry, Aalborg University Hospital, 9000 Aalborg, Denmark; 2 Department of Oncology, Aalborg University Hospital, 9000 Aalborg, Denmark; 3 Department of Basic Medical Sciences, College of Medicine, QU Health, Qatar University, Doha P.O. Box 2713, Qatar; 4 Department of Radiology, Aalborg University Hospital, 9000 Aalborg, Denmark; 5 Department of Biochemistry and Immunology, Lillebælt Hospital, University Hospital of Southern Denmark, 7100 Vejle, Denmark

**Keywords:** small cell lung cancer, extracellular vesicles, Olink, proximity extension assay, PDGF-B, TGF-β1, biomarker, liquid biopsy

## Abstract

**Background**: Small cell lung cancer (SCLC) is an aggressive malignancy with rapid progression and early metastasis, yet it lacks validated biomarkers for early diagnosis and treatment monitoring. Extracellular vesicles (EVs) are nano-sized particles released by cells that carry cargo reflective of their origin, making them promising candidates for liquid biopsy-based biomarker discovery. **Methods**: Plasma-derived EVs were isolated from 29 SCLC patients and 28 healthy controls (HCs) using ultracentrifugation and characterized via nanoparticle tracking analysis (NTA) and transmission electron microscopy (TEM). Proteomic profiling was performed using the Olink^®^ Immuno-Oncology panel. Group differences were analyzed using multivariate models (Boruta, Elastic Net, and partial least squares discriminant analysis). Diagnostic potential was assessed using ROC analysis, and predictive response associations were evaluated by correlating EV protein changes with tumor size reduction (FDR-adjusted Spearman). **Results**: Of the 58 detected proteins, 36 were significantly upregulated in SCLC. PDGF-B, CXCL5, CCL17, EGF, and LAP-TGF-β1 showed good discriminatory performance (AUC 0.95–0.98). Additionally, a two-protein panel (LAP-TGF-β1 and PDGF-B) achieved an out-of-fold AUC of 0.96 (CI 0.89–1.00). NOS3, VEGFR-2, and ANGPT2 levels correlated inversely with tumor reduction after chemotherapy. **Conclusions**: These exploratory findings highlight EV proteomics as a minimally invasive platform for potential SCLC diagnosis and monitoring.

## Simple Summary

Small cell lung cancer (SCLC) is a very aggressive lung tumor with high mortality and a late onset of symptoms. Detecting this disease is usually only possible at late stages, thus limiting treatment options. To date, there are no universally accepted biomarkers available that could aid in diagnosis. Therefore, the aim of this study was to detect changes in the expression levels of proteins carried by extracellular vesicles to pave the way for blood-based biomarkers for SCLC. Using a targeted protein analysis, it was possible to identify a specific pair of proteins that could distinguish healthy people from patients with SCLC. This study shows the potential of extracellular vesicle-derived proteins in the future diagnosis of SCLC.

## 1. Introduction

Small cell lung cancer (SCLC) is a high-grade, aggressive neuroendocrine carcinoma that makes up approximately 15% of all lung cancer cases, leading to over 250,000 deaths worldwide [1,2]. Current diagnostic approaches rely on tissue biopsies and radiological imaging, typically not detecting the cancer before significant disease progression [3]. Despite a good initial response to chemotherapy, most patients experience disease progression within months of treatment, which results in a low five-year survival of approximately 7% [4]. In non-small cell lung cancer (NSCLC), immunotherapies have improved clinical outcomes [5], while SCLC still lacks validated biomarkers to support early detection and treatment response. Although studies have proposed biomarkers for SCLC, none of them have been formally established and clinically useful [6]. This highlights a need for a robust set of biomarkers that could allow for early diagnosis and improve patient outcomes.

Extracellular vesicles (EVs) are membrane-bound nanoparticles that have been documented to be present in various biofluids, including blood. EVs are noted as carriers of various cellular components, such as proteins, genetic material, and lipids, thus making them a useful source for non-invasive cancer diagnostics [7,8,9]. Multiple publications have reported that EV cargo reflects the pathophysiological state of the parent cell [10,11], while others underline that EV protein cargo can be used in a diagnostic and prognostic capacity across many cancers, including SCLC [2,12,13].

Proximity extension assay (PEA) is a highly sensitive, multiplex approach to the simultaneous detection of numerous proteins using minimal sample volumes. This technique uses matched sets of antibodies conjugated to complementary single-strand DNA, thus allowing for protein quantification via PCR amplification with a sensitivity of fg/mL or lower [14,15]. This technique has previously been used to successfully quantify protein cargo from EVs [16].

In this exploratory study, we aim to identify primarily diagnostic and secondarily prognostic biomarkers in follow-up samples for SCLC via larger EV pellets isolated from patient plasma through centrifugation. Using Olink^®^’s (Upsala, Sweeden) Immuno-Oncology panel, we profiled protein signatures from EV samples of SCLC patients compared with healthy controls (HCs) to detect specific cancer- and immune-associated protein profiles with potential clinical utility through univariate and multivariate statistics.

## 2. Methods

### 2.1. Study Population

**Figure 1:**
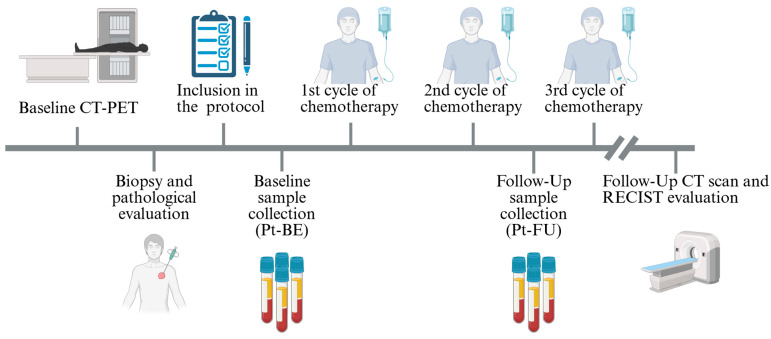
Schematic representation of SCLC-OMICS study protocol. Patients were evaluated with PET-CT and biopsy to determine their eligibility for standard chemotherapy before enrollment. Samples were collected before treatment (Pt-BE) and before the third cycle of chemotherapy (Pt-FU). Response to treatment was evaluated using CT scans after the third cycle of chemotherapy. Created in BioRender. Kristensen, S. (2026) https://BioRender.com/bx1ealc (accessed on 4 February 2026).

This study included 29 patients and 28 HCs in the SCLC-OMICS project at Aalborg University Hospital, Denmark. Patients were diagnosed with SCLC and received treatment between 2022 and 2025 at the Dept. of Oncology, Aalborg University Hospital. A set of blood samples were collected at baseline, before treatment was initiated (Pt-BE), and before the third cycle of treatment as follow-up (Pt-FU). The patients received a standard treatment regime of chemotherapy consisting of Carboplatin and Etoposide as per local guidelines. Routine bloodwork and follow-up CT/CT-PET scans were conducted according to the local treatment guidelines. Inclusion criteria were as follows: age > 18 years; eligibility for standard chemotherapy treatment; measurable disease on CT scans; and histopathologically or cytologically confirmed SCLC. Exclusion criteria were the following: active second malignancy or requirement of treatment for such (except for non-melanoma skin cancer or cervical cancer in situ); concomitant anticoagulation treatment other than acetylsalicylic acid and clopidogrel; and participation in other investigational drug studies. The blood samples from the healthy controls were collected from plasma donors at the Blood Bank at Aalborg University Hospital, Aalborg, Denmark, and these donors were age- and sex-matched to the patient cohort as closely as possible. Inclusion criteria for the HCs were as follows: age > 60 years and no history of cancer, except for non-melanoma skin cancer or cervical cancer in situ. The exclusion criterion was the following: current treatment with anticoagulation medicine aside from acetylsalicylic acid and clopidogrel. Additionally, HCs were asked to answer a health questionnaire regarding their general health, such as their smoking history; diagnosis of diabetes, cardiovascular diseases, or lung conditions; and current pharmacological treatment. They underwent routine biochemical screening for hematology, liver, renal, and hemostatic parameters. This study followed the Declaration of Helsinki, and all participants provided written informed consent. Ethical approval was obtained from the Committee board of Northern Jutland N-20220023. A schematic representation of the study timeline can be seen in Figure 1.

### 2.2. Sample Collection and Clinical Data

Blood was drawn from the antecubital vein using a vacutainer blood collection device with a 21-gauge needle (Vacuette, Greiner Bio-One, Vienna, Austria) and collected in lithium heparin and an EDTA tube for routine analyses and three 6 mL 0.105 M (3.2%) trisodium citrate tubes (BD Vacutainer^®^, Plymouth, UK). Platelet-poor plasma (PPP) was isolated within 30 min of sample collection using double centrifugation at 2500× *g* for 15 min at room temperature, and the plasma was collected 1 cm above the buffy coat from each centrifugation. PPP was pipetted into several vials that were immediately stored at −80 °C until the collection of samples was completed within 2 years. For each of the following analyses, a sample was thawed (only once) before further processing. Data on the included patients with SCLC were collected from hospital records regarding their treatment and medical history, such as staging, smoking history, chemotherapy doses, comorbidities, medicine history (before treatment), and blood biochemical analyses. Study data were collected and managed using REDCap electronic data capture tools hosted at Aalborg University Hospital [17,18].

### 2.3. Biochemical Analysis

Alanine transaminase (ALAT), albumin, alkaline phosphatase, creatinine, potassium, lactate dehydrogenase (LDH), leukocytes, sodium, and bilirubin were analyzed in both patients and HCs on an Alinity ci System (Abbott, North Chicago, IL, USA), while hemoglobin, neutrophils, and thrombocytes were analyzed on Sysmex XN-9100 (Sysmex, Kobe, Japan) at the Department of Clinical Biochemistry, Aalborg University Hospital, Aalborg, Denmark.

### 2.4. Isolation and Preparation of Large EV Pellets

Pelleted EVs were isolated as described previously [16]. In brief, 1.5 mL of PPP underwent centrifugation twice at 20,000× *g* for 1 h at 4 °C in a Multifuge 3 S-R with a fixed-angle rotor (#3332, Heraeus, Hanau, Germany). The resulting EV pellets were washed with 1 mL of 0.23 µm sterile-filtered Dulbecco′s Phosphate-Buffered Saline (DPBS) (Sigma-Aldrich, Burlington, MA, USA) between centrifugations. EV pellets designated for Olink^®^ were resuspended in 10 µL of DPBS and mixed 1:4 with M-PER^TM^ Mammalian Protein Extraction Reagent (Thermo Scientific, Waltham, MA, USA) with Pierce™ Protease Inhibitor Mini Tablets, EDTA-free (Thermo Scientific, USA) and processed according to the manufacturer’s instructions. Samples designated for nanoparticle tracking analysis (NTA) and transmission electron microscopy (TEM) were resuspended in DPBS. All the lysates were stored at −80 °C until analysis.

### 2.5. Nanoparticle Tracking Analysis

NTA was conducted as follows: Isolated EV pellet samples were each measured twice on the ZetaView NTA system (Particle matrix, Inning am Ammersee, Germany). The mean of the two measurements was used for analysis. The system was calibrated using 0.1 µm silica beads (Polysciences, Warrington, PA, USA) as per the manufacturer’s instructions. EV samples were diluted in 0.23 µm sterile-filtered DPBS to reach the ideal particle per frame value (150–250 particles/frame). The instrument settings were as follows: mode—scatter (488 nm); scan positions—11 at 60 fps; camera sensitivity—80; shutter—100; cell temperature—23 °C. The recorded video captures were analyzed with ZetaView Software (8.05.14) with the following settings: min area—10; max area—1000; minimum particle brightness—30. Samples were not blindly measured on the NTA system. We recently estimated the variation in this method [19] as being quite low (coefficient of variation (CV) of 8%) and larger between different samples from the same individual, presenting a value much lower than the between-subject variation.

### 2.6. Transmission and Immunoelectron Microscopy

Pooled isolated EVs were morphologically assessed with transmission electron microscopy (TEM), and the presence of CD9^+^ EVs was confirmed using immunoelectron microscopy (IEM) in the isolated pellets, as previously described [16]. Briefly, for TEM, 5 µL of isolated EVs in DPBS was added to carbon-coated, glow-discharged 400-mesh Ni grids (SPI Supplies, West Chester, PA, USA) for 30 s and excess moisture was removed with filter paper. The grids were stained with one drop of 1% (*w*/*v*) phosphotungstic acid (PTA; pH 7.0, Caspilor AB, Lindingö, Sweden) before drying. For IEM, 5 µL of EV suspension was applied for 30 s to carbon-coated, glow-discharged 400-mesh Ni grids (SPI Supplies, USA); rinsed thrice with PBS; and blocked with 0.5% ovalbumin in PBS (Sigma-Aldrich). Grids were incubated for 30 min at 37 °C with primary monoclonal mouse anti-CD9 (HYB-SSI 382-01, Statens Serum Institut, Copenhagen, Denmark) at 1:50 in 0.5% ovalbumin in PBS and washed three times in PBS. Subsequently, incubation was conducted for 30 min with 10 nm gold-conjugated goat anti-mouse secondary antibody (British BioCell, Cardiff, Wales, UK) at 1:25 in 0.5% ovalbumin in PBS. Samples were again washed thrice with PBS and then incubated thrice for 10 min in 1% cold fish gelatin in PBS (Sigma-Aldrich, USA). IEM samples were stained with 1% (*w*/*v*) phosphotungstic acid and dried as before. Images were captured using a JEM-1010 transmission electron microscope (JEOL, Akishima, Tokyo, Japan) operated at 60 keV and equipped with an electron-sensitive CCD camera (KeenView, Olympus, San Diego, CA, USA).

### 2.7. Western Blot

A Western blot was conducted to characterize the presence of EVs in the isolated pellet samples in essence, as described previously [19]. A pool of four participants per group was used for the Western blot. The isolated EV pellets were resuspended in 25 µL of DPBS, and 10 µL of each sample was used for the pool. A total of 20 µL of the sample and sample buffer (2× Lameli sample buffer, Bio-Rad, USA) in a 1:1 ratio was added to each well. Proteins were separated using Mini-PROTEAN^®^ TGX™ 4–15% gels (Bio-Rad, Hercules, CA, USA) for 5 min at 50 volts and then at 150 volts for 45 min under non-reducing conditions. After protein separation, they were transferred onto a 0.45 µm PVDF blotting membrane (Bio-Rad, Hercules, CA, USA) for one hour under 100 volts and subsequently blocked in 5% (*w*/*v*) skimmed milk Tris–Glycine buffer (blocking buffer). The primary antibodies were as follows: mouse monoclonal anti-human CD9 (HYB-SSI 382-01, Statens Serum Institut, Copenhagen S, Denmark) and rabbit polyclonal anti-AIP1/Alix (Cat. #ABC40) (EMD, Millipore, Darmstadt, Germany) diluted 1:1000 in blocking buffer. The membranes were incubated overnight with primary antibodies at 4 °C. Subsequently, the membranes were incubated with secondary horseradish peroxidase-conjugated goat anti-mouse antibody (Dako, Glostrup, Denmark) and goat anti-rabbit antibody (Cat. #170-5046) (Bio-Rad, Hercules, CA, USA) for two hours at room temperature. The membranes were developed using Lumi-Light Plus Western blotting substrate detection reagent (Roche, Basel, Switzerland) and imaged using a PXi 4 system (Syngene, Cambridge, UK) equipped with GeneSys software version 1.5.4.0 (Syngene, UK). The negative control was sample buffer in D-PBS.

### 2.8. Proximity Extension Assay

Protein measurements were performed using the Olink^®^ Immuno-oncology panel, which is a commercially available multiplex proximity extension assay (Olink Proteomics, Uppsala, Sweden) conducted by BioExpedia a/s, Aarhus, Denmark. To ensure comparability across all EV pellet samples, the protein content was first measured using a Pierce™ bicinchoninic acid assay kit (Thermo Scientific, USA) according to the manufacturer’s instructions, and the samples were subsequently diluted to the same protein concentration. All samples were randomized on the plate according to the manufacturer’s recommendations. Additional details about this method were provided previously [16]. In brief, two sets of antibody adaptors conjugated to a specific ssDNA can attach to specific peptide sequences on the target protein. The proximity of the DNA strands allows for hybridization and the subsequent amplification of the product using a qPCR. When a qPCR signal rises sufficiently above the background, cycle threshold values (Ct-values) are registered and used for relative protein quantification as log_2_-transformed Normalized Protein Expression (NPX) values. These values were calculated in the following manner:

Correction of extension Ct control to sample Ct: ${Ct}_{Analyte}-Ct_{Extension\;Control}=dCt_{Analyte}$ . Correction against Inter-Plate control: $d{Ct}_{Analyte}-{dCt}_{Inter-plate\;Control}={ddCt}_{Analyte}$ .

Adjustment against a correction factor: $Correction\;factor-{ddCt}_{Analyte}=NPX_{Analyte}$ .

The Intra-Assay CV Distribution for proteins was as follows: <5% (*n* = 42), ≥5–<10% (*n* = 37), ≥10–<15% (*n* = 2), ≥15% (*n* = 6), N/A (*n* = 5).

### 2.9. Treatment Response Correlations

Two patients did not receive a follow-up CT due to either patient death or the cessation of treatment and thus were not used for the correlation analysis, resulting in a total of 27 patients. All participants received at least 75% of the planned dose of Carboplatin (5 AUC i.v.) and 75% of Etoposide (200 mg/m^2^ p.o. or 120 mg/m^2^ i.v.,) for at least 2 cycles of chemotherapy. Response to treatment was estimated according to the RECIST 1.1 criteria [20], consisting of the percent-wise difference in change in the Sum of Longest Diameters (SLD) between baseline and follow-up (%∆SLD) . %∆SLD was correlated to the expression levels (NPX) of proteins at baseline and follow-up using a Benjamini–Hochberg (BH)-corrected Spearman correlation. The correlations were interpreted as follows: ρ < 0: the protein level increases with tumor regression; ρ > 0: the protein level decreases with tumor regression. Protein expression levels were divided into four quartiles (Q1–Q4). The quartiles were defined based on the distribution of ΔNPX (expression levels), with Q1 representing the lowest (most negative) and Q4 the highest changes. For each protein, the distribution of percentage change in tumor SLD (%ΔSLD) was then compared across quartiles, and Q1 and Q4 were specifically compared using either a Wilcoxon rank-sum test or a two-sample *t*-test, as appropriate.

### 2.10. Data Processing and Statistical Analysis

Data were presented as the mean ± standard deviation (SD) or the median with the interquartile range (IQR) depending on the Gaussian distribution assessed with the Kolmogorov–Smirnov test of normality. NPX data was first filtered to remove proteins under the assay’s limit of detection (LOD). The proteins were then filtered to remove those with >30% missing values. The remaining proteins were used for downstream analysis. To elucidate differences between groups, comparisons were conducted using Student’s *t*-test and a one-way ANOVA, both with BH correction. Nonparametric data was tested using the Wilcoxon ranked-sum test or Kruskal–Wallis test, also with BH correction. A volcano plot was generated to visualize significantly enriched proteins with >1 log FC and *q* < 0.05. To investigate the diagnostic potential of the proteins of interest, single biomarker assessments were conducted for the top five most differentially expressed proteins between HC and Pt-BE based on the fold change (NPX values). Receiver operating characteristic (ROC) curves were generated using the pROC package (v. 1.4), with the sensitivity and specificity thresholds being set by Youden’s J.

An unsupervised principal component analysis (PCA) and a supervised Partial Least Squares Discriminant Analysis (PLS-DA) was performed on centered and scaled data was first conducted to assess trends in the whole dataset, and afterwards, three machine learning models were tested to identify the most optimal protein signature to distinguish baseline SCLC patients (Pt-BE) from HCs.

**Figure 2:**
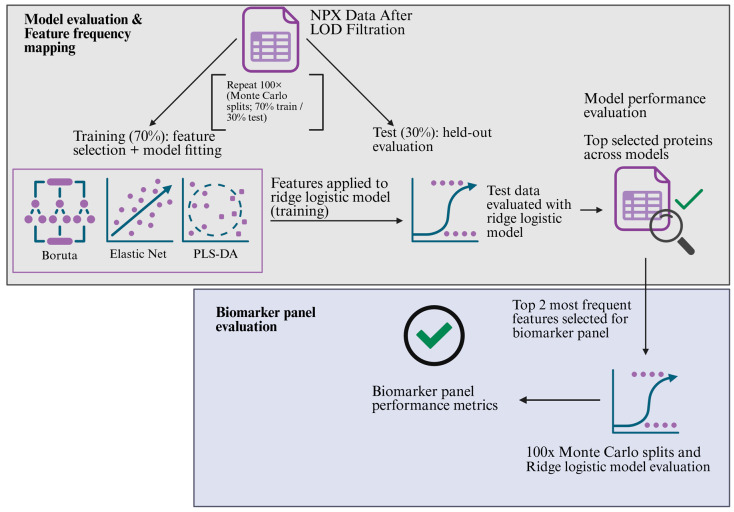
A schematic illustration of model evaluation, feature frequency mapping, and biomarker panel evaluation. Model evaluation: Data is split at 70/30% for the training and test sets 100 times using Monte Carlo splits. Feature selection was conducted with Boruta, Elastic Net, and PLS-DA. Each model selects the five best-performing proteins that contributed the most to group separation. This information is then used to establish a ridge logistic model per algorithm. Each feature selector-based ridge logistic model is then applied to the test set, i.e., previously unseen data for the model. Performance is converted to ROC scores, and the probabilities of the selected proteins are kept. Biomarker panel evaluation: Across the three models, a consensus panel is selected by choosing the top two most selected proteins across them. The two-protein biomarker panel is then evaluated using a ridge logistic model on a new set of 100 Monte Carlo splits, thus allowing for the computation of panel performance. Created in BioRender. Krzyslak, H. (2026) https://BioRender.com/iz77lll (accessed on 4 February 2026).

Within iteration modeling:. First, using the caret package (v.7.0-1), a 100-iteration Monte Carlo cross-validation (MCCV) 70/30 split of the NPX data was used to divide the data into a training and test set, respectively. Feature selection was performed within each MCCV training split, and missing values were handled using median imputation, with the same imputation values applied to the corresponding test split. Three selection models (Boruta, Elastic Net, and PLS-DA) were used. For Boruta feature selection, the Boruta (v. 9.0.0) R package was used for 500 iterations, with a confidence level of 0.01 and Bonferroni correction. Features were ranked by median importance, and the top 5 features were selected for model testing. For elastic net, the glmnet (v. 4.1-10) R package was used, with the alpha level set to 0.5 and the measurement type chosen as AUC. Features with non-zero coefficients were retained, and the top five coefficients were chosen. Finally, for PLS-DA, the mixOmics (v. 6.30.0) R package was used with the maximum number of components set to 5 and scaling enabled. The number of latent variables (LVs) was chosen using repeated M-fold CV on the training set with 5 folds and 10 repeats. The features were ranked by VIP, and the top five were kept. PLS component scores derived from these proteins were used as predictors in the ridge logistic model. For all models, a ridge-penalized logistic regression classifier was used on the training data. Prior to this, the training data were z-scored using the mean and SD. The ridge penalty parameter was selected within each training split via cross-validation using the 1-SE rule (λ.1se). Features in the test set were similarly standardized, and predictions were made using the same penalty parameters without further tuning. Across-iteration model evaluation and biomarker panel derivation: Model performance was evaluated by (i) the distribution of the held-out test set AUC across repeats and (ii) a pooled out-of-sample AUC computed by aggregating test set predictions across repeats, taking the per-subject median predicted probability over all repeats in which the subject was held out, and calculating the AUC on these per-subject medians. To establish the biomarker panel, the selection frequencies of individual proteins across all three models and MCCV repeats were assessed. Stable proteins were defined as those selected in ≥60% of repeats. The two-protein panel was defined as the intersection of the three models’ stable sets. The panel was evaluated through a fresh set of MCV splits and with the same ridge logistic workflow and OOF aggregation as described above. A schematic overview of the biomarker selection loop can be seen in Figure 2. All analyses were performed in R-studio version 2024.09.1 using R version 4.4.2.

## 3. Results

### 3.1. Participant Characteristics

Patient and HC cohorts were matched by age and sex (Table 1). Most of the patients presented with a disseminated (IV) stage of SCLC as their diagnosis and had a low Charlson comorbidity index (CCI:1) and a performance status (PS) of 1 (Table 1). A total of 97% of the patients were either current or previous smokers with a median of 40 packyears, in contrast to 46% of HCs with a median of 10 packyears. Some patients were characterized as having elevated levels of lactate dehydrogenase (LDH), leukocytes, neutrophils, and thrombocytes in comparison with HCs, with the highest changes observed for LDH. Decreased hemoglobin values were also observed in SCLC patients (Table 2). All biochemical parameters for the HCs were within the reference range. None of the HCs were diagnosed with any chronic medical condition.

### 3.2. Characterization of Isolated Extracellular Vesicles

**Figure 3:**
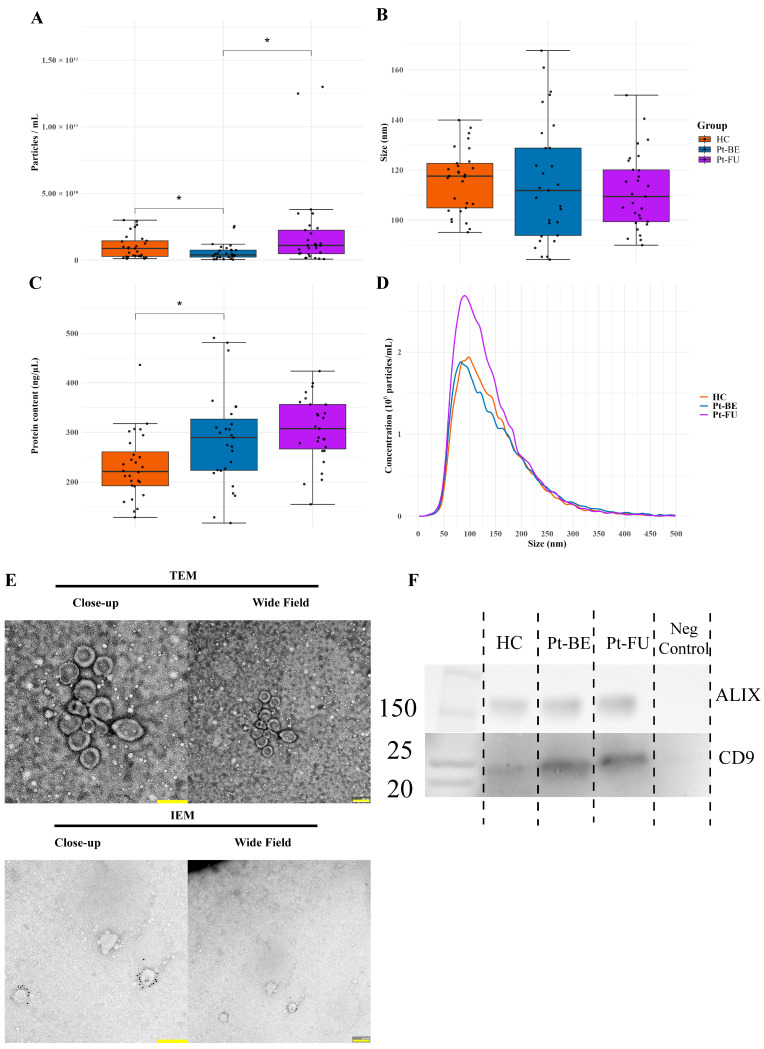
Extracellular vesicle characterization. (**A**) Nanoparticle tracking analysis (NTA) of particle concentration (particles/mL). (**B**) NTA size distribution (mean/median diameter) and representative distribution curves. (**C**) Total protein concentration of EV samples prior to Olink analysis. (**D**) Size/distribution curve of isolated particles measured in all samples across all three groups with NTA. (**E**) Representative transmission electron microscopy (TEM) and immunogold electron microscopy (IEM) images confirming vesicular morphology and CD9+ particles. (**F**) Western blot shows presence of CD9 and Alix-positive extracellular vesicles in all three groups. Scale bars: 200 nm. HCs: healthy controls; Pt-BE: patients at baseline (before treatment); Pt-FU: patients at follow-up (after 3rd round of chemotherapy). *p* < 0.05 *.

NTA showed a significant difference in particle concentration in samples between the HC cohort and Pt-BE (*p* = 0.041) and between cohorts Pt-BE and Pt-FU (*p* = 0.020) (Figure 3A). There were also significant changes in protein concentration between HCs and Pt-BE (*p* = 0.019) with a higher protein level in the Pt-BE group compared to HCs before normalization (Figure 3B). Particle distribution demonstrated that there were mainly larger EVs (>100 nm), but a larger subset of particles <100 nm was found in Pt-BE compared to the HC and Pt-FU groups, although the overall mean size differences in the particles were not significant between the groups (Figure 3B,D), thus indicating differences in measured particle concentrations but not the size of the particles. Some differences in group sample protein concentration pre-equalization for Olink^®^ analysis were observed (Figure 3C) Electron microscopy images showed clear vesicular structures, and immune gold labeling confirmed the presence of CD9^+^ EVs, thus indicating the isolation of intact EVs within the appropriate size range of approximately 100–200 nm (Figure 3E). A Western blot demonstrated the presence of CD9- and Alix-positive EVs in all preparations (Figure 3F). We previously showed that these pellets are somewhat contaminated with lipoproteins (apoB) [19].

### 3.3. Detection of Plasma-Derived Extracellular Vesicles via Proximity Extension Assay

**Figure 4:**
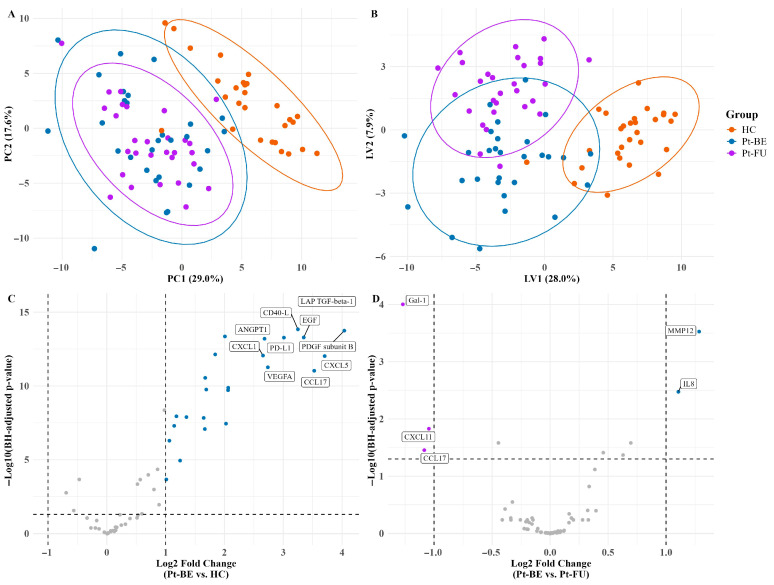
Multivariate models distinguish SCLC patients from healthy controls. (**A**) Principal component analysis (PCA) on centered and scaled NPX values; ellipses indicate 95% data coverage. Axes show variance explained. (**B**) Partial least squares discriminant analysis (PLS-DA) with first two latent variables (LVs); axes show variance explained. (**C**,**D**) Volcano plots for (**C**) Pt-BE vs. HC and (**D**) Pt-FU vs. Pt-BE. Points are colored in terms of direction of change; *p*-values are Benjamini–Hochberg-adjusted (*q*-values), and ΔNPX > 1 (log2) threshold corresponds to ≥2-fold change on linear scale. Colors indicate which group protein is upregulated/downregulated in. HCs: healthy controls; Pt-BE: patients at baseline (before treatment); Pt-FU: patients at follow-up (after third round of chemotherapy). Grey dots signify non-significant proteins less than one log2 fold change after BH correction.

The isolated EVs were analyzed with the Target 96 Immuno-Oncology panel. The panel confirmed the presence of 58 of 96 proteins after filtration. A full list of all identified proteins and their NPX values after filtration can be found in the Supplementary Materials (Table S1). The PCA shows separation between the HC and Pt-BE groups, thus indicating a difference in proteome expression, while the groups Pt-BE and Pt-FU show some overlap (Figure 4A). The two principal components (PCs) explain 46.6% of the variation in the data. The PLS-DA model shows a clearer separation of the three groups, thus suggesting proteome expression changes between all the groups (Figure 4B), with the first two latent variables (LVs) explaining 35% of the variation. The volcano plots show 25 upregulated proteins with higher expression levels in the Pt-BE group compared to HCs (Figure 4C) and three downregulated proteins. The follow-up group Pt-FU has two upregulated proteins compared to Pt-BE (Figure 4D).

### 3.4. Diagnostic Biomarker Assessment

**Figure 5:**
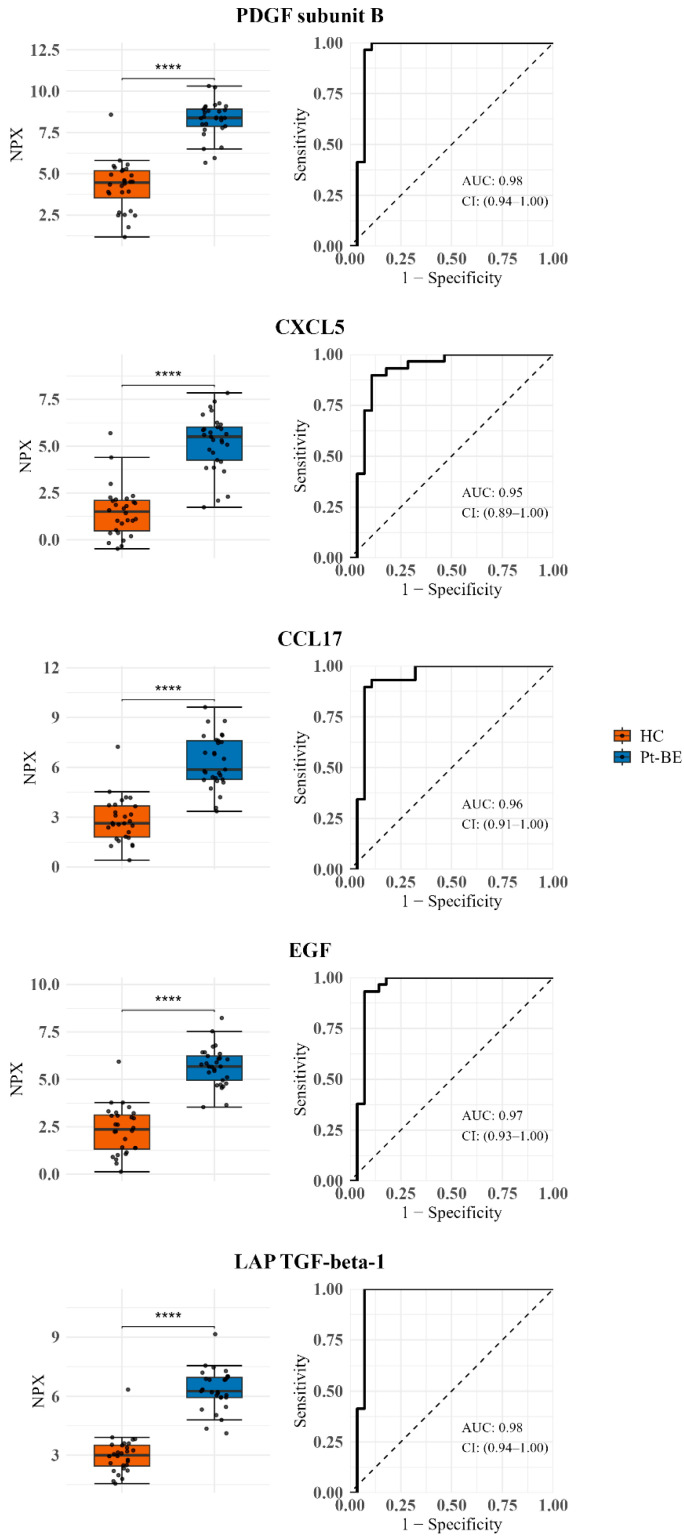
Top univariate analysis-determined proteins exhibit strong discrimination of baseline Pt-Be vs. HC. Boxplots (**left**) show NPX distributions for five most upregulated proteins in Pt-BE compared with HCs (ranked by ΔNPX). Receiver operating characteristic (ROC) curves (**right**) report AUC with 95% confidence intervals. HCs: healthy controls; Pt-BE: patients at baseline (before treatment). *p* < 0.0001 ****.

First, to assess the discriminatory properties of single protein biomarker candidates, the top five most upregulated proteins were selected. The corresponding boxplots and ROC curves for each protein comparing HC and baseline samples are presented in Figure 5. PDGF subunit Beta showed the highest AUC of 0.98 with a sensitivity and specificity of 0.97 and 0.96, respectively. All the significant proteins detected in the dataset and ROC curves for significant proteins above 1 delta fold change can be seen in Supplementary Tables S2 and S3.

### 3.5. Model Performance and Biomarker Panel Candidates

Filtered proteins were assessed as potential biomarkers through three separate models: Boruta, Elastic Net, and PLS-DA (for further details, see Section 2). Each model was configured to retain the top five most important proteins selected independently by the used algorithm, and performance (the overall capacity of each model to separate HCs from Pt-BE) was evaluated. The top five most frequent subsets of markers selected by the models and their selection probability are shown in Table 3.

The performance characteristics of each of the models based on their five selected proteins were evaluated (Table 4). Overall, all three algorithms performed well, with the PLS-DA and Boruta models performing the best of the three (AUC: 1; IQR: 0.93–1). The Boruta and Elastic Net models showed identical performance with an AUC of 0.97.

Between all three models, LAP TGF-beta-1 and PDGF subunit B were selected for a consensus panel. The performance of this panel was evaluated, and good performance characteristics were shown, with a median AUC of 1 (IQR: 0.96–1) and pooled OOF AUC of 0.96 (CI: 0.89–1).

### 3.6. Exploratory Treatment Response Biomarker Analysis

**Figure 6:**
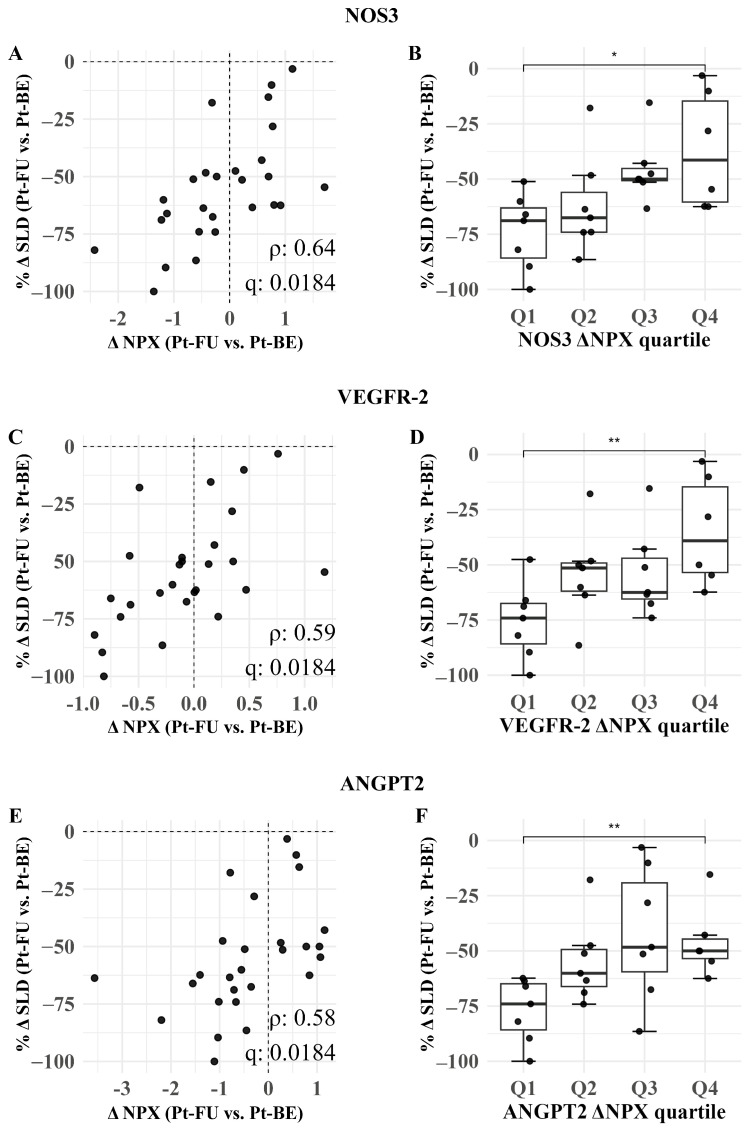
Changes in angiogenesis-associated EV proteins correlate with tumor shrinkage following chemotherapy. (**A**,**C**,**E**) show Spearman correlations between ΔNPX (Pt-FU and Pt-BE) and %ΔSLD with Benjamini–Hochberg-adjusted *q*-values; (**B**,**D**,**F**) show %ΔSLD distributions across ΔNPX quartiles (Q1–Q4) with Q1 vs. Q4 comparisons. Pt-BE: patients at baseline (before treatment); Pt-FU: patients at follow-up (after the 3rd round of chemotherapy. Dashed lines indicate the zero-change reference (ΔNPX = 0 and %ΔSLD = 0). *p* < 0.05 *, *p* < 0.01 **.

To explore whether treatment response was reflected in within-patient protein dynamics, we correlated the percent change in tumor burden (%ΔSLD; follow-up vs. baseline) with the corresponding change in protein expression (ΔNPX = follow-up - baseline).Three statistically significant correlations were found (Figure 6). All three proteins showed no significant changes, but patients with greater tumor shrinkage showed larger decreases in NOS3, comparing baseline and follow-up (ρ = 0.64; *q* = 0.018), with a significant quartile change from Q1 to Q4 (*q* = 0.026). Similarly, VGFR-2 showed a moderate correlation ρ = 0.59 with tumor shrinkage with an adjusted *q* = 0.018, while ANGPT2 had the lowest correlation ρ = 0.58 with an adjusted *q* = 0.018.

## 4. Discussion

In this exploratory, single-center cohort study, we demonstrated that large EV pellets isolated from the plasma of patients with SCLC carry distinctive protein signatures with high potential diagnostic value. Using targeted Olink^®^ Immuno-Oncology PEA, we identified a two-protein panel, consisting of LAP TGF-β1 and PDGF subunit B, that robustly distinguished SCLC patients from healthy controls with high accuracy (OOF AUC of 0.96). These proteins were repeatedly selected by three independent feature selection methods, thus highlighting their robustness. For Olink analysis, some samples did have some variation in protein concentration. Thus, to ensure comparability, the samples were diluted to a fixed protein concentration. This choice could potentially risk underestimating (if the SCLC samples have a high content of other proteins) or overestimating (if the SCLC samples have a low content of other proteins) some true differences in specific protein expression in the groups. Thus, to ensure the reliability of our results, for each sample, we used the measured pre-adjustment protein concentration to reverse normalization and repeated the analysis for significant biomarkers. This repetition resulted in slightly more significant markers in the reversed dataset (49 vs. 36), but, nevertheless, a 91.6% overlap between the two sets. The reversal of normalization did not affect the significance of the proteins reported on. Even though the variation in protein content could have influenced protein significance, our analysis suggests a limited impact.

In our cohort, smoking exposure differed substantially between controls and patients (ever-smokers: 46% in controls vs. 97% in patients; median exposure: 10 vs. 40 packyears). Small cell lung cancer is strongly associated with cigarette smoking, and never-smokers constitute a small minority of SCLC cases in large clinical studies [21,22]. Consequently, identifying older, heavily exposed controls without comorbidities can be challenging, as smoking is associated with multimorbidity and cardiopulmonary disease burden [23,24]. Given this imbalance, smoking represents a potential confounder for case–control comparisons. We therefore conducted sensitivity analyses of the biomarker panel: (i) we repeated the panel evaluation after restricting the cohort to ever-smokers only, and panel discrimination remained high in this sub-analysis (pooled OOF AUC = 1.00; control ever-smokers *n* = 13; patients *n* = 28), although the small number of smoking controls warrants cautious interpretation. (ii) To assess whether the panel behaves as a smoking proxy, we tested whether the panel could classify ever-smoking within controls, but it did not (AUC 0.50). Overall, there are relatively strong indications suggesting that the panel signal is not simply a proxy for ever-smoking among healthy individuals, but the effect of smoking cannot be fully ruled out.

Isolating contaminant-free extracellular vesicle preparations is an ongoing challenge [25]. Many currently established techniques have a degree of contamination in EV preparations, including high-speed centrifugation [26]. These preparations typically include a degree of apolipoprotein B (ApoB) present on low/very-low-density lipoproteins, which can overlap with the size and density of EVs [27]. Previous studies have found a degree of this contamination in EV pellets isolated at 20,000× *g* [19,28]. This study, however, uses a targeted proteomic approach via O-link^®^, which is tailored to target specific signals related to inflammation and oncology. Thus, it is not very likely that the signals in this study are a result of lipoparticle contamination. Further, we recently published a study analyzing short-term diurnal composition (pre-/postprandial EV pellet protein cargo composition) and found a lack of significantly different *q*-values [19]. Our aim was to establish a set of biomarkers for early detection, but most of the patients included were stage IV at diagnosis. Therefore, the identified panel may be regarded as a discriminator of advanced SCLC versus controls, and its applicability to early-stage SCLC remains uncertain. Future studies will focus on exploring these aspects of the earlier detection of patients with SCLC.

Several individual EV-associated proteins also showed high discriminatory power (AUC ≥ 0.95), including PD-L1, CCL17, CXCL5, and EGF. These top-performing markers show patterns that are biologically plausible given their established roles in angiogenesis, stromal activation, and immunoregulatory signaling. EV-associated PD-L1, which was previously linked to immune evasion and treatment response in other cancers, further supports the relevance of EV cargo in SCLC biology. PDGF-B/PDGFR signaling contributes to angiogenesis and stromal activation in lung cancer, with several studies linking tumor or stromal PDGF axis expression to aggressive behavior and poor prognosis in NSCLC [29,30]. Elevated PDGF-B levels in tumors correlate with advanced disease stage and shorter survival time. The identification of EV-associated PDGF-B as a key discriminatory marker in our cohort suggests that EV cargo may capture this pro-angiogenic signaling axis in SCLC. Similarly, TGF-β is a multifunctional cytokine with complex roles in tumor suppression and progression. It is secreted as an inactive complex bound to the latency-associated peptide (LAP), and EVs have been identified as carriers of this latent form [31,32,33]. Altered TGF-β signaling has repeatedly been associated with aggressive phenotypes and immune/stromal remodeling in lung cancer, including NSCLC, although the direction and clinical implications of circulating TGF-β measures can vary by cohort and methodology [34]. The presence of LAP TGF-β1 associated with EVs may indicate systemic immunosuppressive signaling and microenvironmental remodeling that favors tumor persistence. Mechanistic studies suggest that EV-associated LAP TGF-β1 acts as a paracrine regulator, promoting fibroblast activation and immune suppression [31,33]. Murai et al. demonstrated that EZH2 silences the TGF-β type II receptor (TβRII), modulating Smad signaling and reducing the pro-apoptotic, tumor-suppressive function of TGF-β in SCLC models. Restoring TβRII re-enabled TGF-β-induced apoptosis and suppressed tumor growth in vivo [35]. Therefore, the elevated levels of EV-associated LAP-TGF-β1 observed in our cohort may reflect the extracellular immunomodulatory functions of TGF-β rather than its canonical tumor-suppressive role within cells.

Other top-performing markers highlight complementary biological pathways relevant to lung cancers, such as PD-L1. PD-L1 is an immune checkpoint protein that binds PD-1 on activated T cells, delivering an inhibitory signal that enables tumor cells to evade immune-mediated destruction. Elevated exosomal PD-L1 levels have been associated with disease burden in melanoma and treatment outcomes for immunotherapy for NSCLC [36,37]. In lung cancer models, EV-derived PD-L1 has been shown to reduce interferon-γ secretion and promote tumor growth. Clinically, correlations were previously found that linked the levels of EV PD-L1 in circulation to the levels detected in stained tumor samples, with similar correlations found for T-cell infiltration and clinicopathologic features [38]. Importantly, these correlations were observed only for EV-associated PD-L1, not for free plasma PD-L1, thus underscoring the biological relevance of EV cargo [38,39]. Transcriptomic analysis links CXCL5 to increased neutrophil infiltration, advanced disease stage, and poor outcomes in lung adenocarcinoma [40,41]. Deng et al. further emphasized CXCL5’s role in angiogenesis within the tumor microenvironment, facilitating immune evasion through neutrophils and supporting tumor proliferation through the PI3K/AKT pathway [42]. While PD-L1 is commonly used to make decisions regarding immunotherapy in NSCLC, its expression patterns and predictive value are less consistent in SCLC, in which reported associations vary by histological subtype and whether PD-L1 is assessed on tumor cells versus immune infiltrates. Accordingly, any relationship between PD-L1 levels, immune evasion, and clinical outcome in SCLC should be interpreted cautiously and in the context of tumor-infiltrating immune cells [43].

CCL17 participates in the CCL17/CCL22–CCR4/CCR8 chemokine axis implicated in regulatory T-cell recruitment and tumor-associated immune suppression; in SCLC, this axis has been described in the context of TAM-derived CCL17/CCL22 signaling. The elevated EV-associated CCL17 levels observed here may therefore reflect the systemic engagement of immunomodulatory chemokine signaling in SCLC [44]. EGF/EGFR axis signaling is central to proliferative programs in lung cancer biology. In an oncologic cohort including pulmonary neoplasms, baseline serum EGF differed significantly from that of healthy controls [45]. These observations collectively support the biological plausibility of our EV-derived biomarkers, which capture immune, angiogenic, and proliferative hallmarks of SCLC pathophysiology.

Exploratory response analysis revealed that reductions in NOS3, VEGFR-2, and ANGPT2 moderately correlated with tumor size regression (Spearman ρ = 0.64, 0.59, and 0.58, respectively), which is consistent with their established roles in angiogenesis and poor outcomes in lung cancer. NOS3 (eNOS) participates in nitric oxide-related vascular biology. In lung cancer, systemic NO biology is frequently altered: exhaled NO is increased in patients with primary lung cancer, and higher circulating nitrite/nitrate (NOx) levels have been associated with poorer survival in late-stage disease. Consistent with the anti-apoptotic role of NO signaling, higher tumor iNOS/NO levels have been linked to reduced caspase-3 activity in human NSCLC tissue [46,47,48,49,50]. VEGFR-2 is similarly associated with aggressive tumors, and high VEGFR-2 expression levels have been linked to shorter survival in NSCLC patients treated with Osimertinib [51]. In SCLC trials using an Etoposide/platin backbone with serial plasma sampling at baseline and pre-cycle 4, soluble VEGFR-2 was measurable longitudinally, though changes in angiogenic factors (including sVEGFR-2) were not strong standalone predictors of response, thus highlighting the need for further validation of VEGFR-2 dynamics as a response biomarker in Etoposide/platin-treated patients with SCLC [52]. Lastly, elevated circulating ANGPT2 levels are associated with more advanced disease biology in lung cancer, including higher levels with advanced stage and distant metastasis in NSCLC and worse overall survival in univariate analyses in NSCLC cohorts. In SCLC, the baseline ANGPT2 level is an independent negative prognostic marker that increases at progression [53,54,55]. Together, prior clinical studies support ANGPT2 as a circulating angiogenesis marker associated with advanced disease and adverse outcomes and show that the level of serum ANGPT2 increases at progression in SCLC, which is consistent with our observation that the level of EV-associated ANGPT2 decreases in patients with greater tumor regression. For NOS3 and VEGFR-2, the observed response-linked EV changes are biologically plausible, given their known roles in vascular biology, but require external longitudinal validation to establish their utility as treatment response biomarkers.

This study has several strengths, including the use of a highly sensitive targeted proteomic approach and multiple selection methods paired with a shared classifier to isolate the effect of feature choice. The final consensus panel was supported by convergent evidence, providing additional insights into SCLC biology.

However, there are important limitations to consider. Firstly, the relatively small population sample size underlines the exploratory nature of this study, limiting power and stage-specific analysis. Secondly, the use of healthy blood donors as the control group maximizes the signal-to-noise ratio but may overestimate real-world performance in symptomatic populations with other diseases (e.g., NSCLC or benign lung conditions), thus meaning that the described markers may not be specific to SCLC. Thirdly, patients were included in the disseminated stage due to the insidious nature of SCLC, thus limiting the ability of the presented biomarkers to detect SCLC at an early stage. Fourth, the endpoint for tumor reduction (follow-up CT scan) occurred one to two months after sample collection, thereby potentially masking the full treatment effect on protein levels and inflating correlation strength compared to simultaneous sampling and imaging. Fifth, the lack of an external validation cohort limits generalizability. Sixth, most patients responded well to treatment, thus making a stratified correlation analysis based on RECIST categories impossible. Finally, while the Olink^®^ platform offers high sensitivity, its targeted nature restricts analysis to predefined proteins; untargeted EV proteomics could reveal additional relevant markers.

## 5. Conclusions

Using the Olink^®^ Immuno-Oncology PEA panel, several highly upregulated proteins were found in the SCLC patient group. With three machine learning algorithms, we developed a compact preliminary diagnostic panel consisting of PDGF subunit B and LAP TGF-β1, achieving an OOF AUC of 0.963. The findings reinforce the growing evidence that EV cargo reflects clinically relevant aspects of tumor–host biology and can be leveraged through minimally invasive approaches. Furthermore, the observed correlations between NOS3, VEGFR-2, and ANGPT2 and tumor reduction highlight the potential of EV proteomics as a real-time indicator of treatment response. Future research should focus on validating these findings in larger, independent cohorts and exploring the utility of NOS3, VEGFR-2, and ANGPT2 as predictive markers for therapy response or disease relapse.

## Figures and Tables

**Table 1:** Participant characteristics and selected clinical parameters from the patient group. ^a^ mean ± SD.

**Study Demographics**				
		Patients (*n* = 29)		HC (*n* = 28)
Age (years) ^a^		67.5 (±6.8)		66.6 (±3.2)
Male/Female (*n*)		15/14		13/15
**Patient Clinical Parameters**				
Overall Stage	-	II: 2 (7%)	III: 3 (10.2%)	IV: 24 (82.8%)
Charlson Comorbidity Index	(0): 10 (34%)	(1): 14 (48%)	(≥2): 5 (17%)	-
WHO Performance Status	(0): 5 (17.2%)	(1): 13 (44.8%)	(2): 8 (27.6%)	(3): 3 (10.3%)

**Table 2:** Biochemical parameters. ^a^ mean ± SD, ^b^ median with IQR. M/F: reference interval for males/females. HCs: healthy controls. Pt-BE: patients at baseline (before treatment).

Biochemical Parameters			
Analyte	Patients (Pt-BE)	HC	Reference
Alanine aminotransferase (U/L) ^b^	23.5 [13.0–44.2]	20.0 [18.0–26.0]	10.0–70.0
Albumin (g/L) ^b^	36.0 [32.0–40.0]	43.0 [42.0–44.0]	34.0–45.0
Alkaline phosphatase (U/L) ^b^	95.0 [76.0–118.2]	78.0 [69.0–93.0]	40.0–120.0
Creatinine (µmol/L) ^b^	68.0 [56.0–83.0]	77.0 [68.0–87.0]	M: 60.0–105.0; F: 45.0–90.0
eGFR (mL/min/1.73 m^2^) ^b^	90.0 [77.0–90.0]	89.0 [81.0–91.0]	>60.0
Hemoglobin (mmol/L) ^a^	8.4 (±1.1)	8.9 (±0.5)	M: 8.3–10.5; F: 7.3–9.5
Potassium (mmol/L) ^b^	3.9 [3.7–4.1]	3.9 [3.7–4.0]	3.5–4.6
Lactate dehydrogenase (U/L) ^b^	289.0 [211.0–437.5]	208.0 [174.5–217.5]	115.0–255.0
Leukocytes (×10^9^/L) ^a^	9.8 (±3.2)	6.2 (±1.4)	3.5–10.0
Sodium (mmol/L) ^a^	139.0 (±3.1)	139.5 (±1.6)	137.0–145.0
Neutrophils (×10^9^/L) ^a^	7.3 (±3.1)	3.7 (±1.1)	2.0–7.0
Bilirubin (µmol/L) ^b^	8.5 [6.0–12.2]	9.0 [7.0–10.5]	5.0–25.0
Thrombocytes (×10^9^/L) ^b^	368.0 [299.0–411.0]	260.0 [209.0–286.5]	145.0–350.0

**Table 3:** Likelihoods of protein selections for each model (Boruta, Elastic Net, and PLS-DA), i.e., number of times selected out of 100 runs, as described in Methods.

Top 5 Most Selected Proteins for Each Model		
Feature	Times Selected/100	Model
LAP TGF-beta-1	99	Boruta
PDGF subunit B	98	Boruta
PD-L1	67	Boruta
CD40-L	66	Boruta
ANGPT1	56	Boruta
LAP TGF-beta-1	86	Elastic Net
CD40-L	57	Elastic Net
PDGF subunit B	53	Elastic Net
PD-L1	24	Elastic Net
CASP-8	22	Elastic Net
LAP TGF-beta-1	100	PLS-DA
PDGF subunit B	98	PLS-DA
CD40-L	98	PLS-DA
EGF	62	PLS-DA
PD-L1	58	PLS-DA

**Table 4:** Performance of three chosen models. ^a^: IQR.

Model Performance—Per-Split AUC and Pooled OOF AUC		
Model	Median AUC	Pooled OOF AUC
Boruta	1 (0.93–1) ^a^	0.96
Elastic Net	0.984 (0.93–1) ^a^	0.97
PLS-DA	1 (0.93–1) ^a^	0.96

## Data Availability

Data will be provided upon request.

## Supplementary Materials

The following supporting information can be downloaded at: https://www.mdpi.com/article/10.3390/cancers18040580/s1, Table S1: A full list of all identified proteins and their NPX values after filtration; Table S2: Summary of all significant proteins detected in the filtered dataset, with expression levels, raw and corrected *p*-values; Table S3: Summary of all ROC curves for significant proteins between CN and BE, including AUC and predictive assessment and 95% CI.

## References

[1] Gazdar A.F., Bunn P.A., Minna J.D. (2017). Small-cell lung cancer: What we know, what we need to know and the path forward. Nat. Rev. Cancer.

[2] Krpina K., Vranić S., Tomić K., Samaržija M., Batičić L. (2023). Small Cell Lung Carcinoma: Current Diagnosis, Biomarkers, and Treatment Options with Future Perspectives. Biomedicines.

[3] Sumon M.S.I., Malluhi M., Anan N., AbuHaweeleh M.N., Krzyslak H., Vranic S., Chowdhury M.E.H., Pedersen S. (2024). Integrative Stacking Machine Learning Model for Small Cell Lung Cancer Prediction Using Metabolomics Profiling. Cancers.

[4] Simpson K.L., Rothwell D.G., Blackhall F., Dive C. (2025). Challenges of small cell lung cancer heterogeneity and phenotypic plasticity. Nat. Rev. Cancer.

[5] Tian J., Shi Z., Zhao L., Liu P., Sun X., Long L., Zang J., Xiao J. (2024). Revolutionizing NSCLC Treatment: Immunotherapy Strategies for EGFR-TKIs Resistance. Clin. Respir. J..

[6] Pedersen S., Jensen K.P., Honoré B., Kristensen S.R., Pedersen C.H., Szejniuk W.M., Maltesen R.G., Falkmer U. (2022). Circulating microvesicles and exosomes in small cell lung cancer by quantitative proteomics. Clin. Proteom..

[7] Halvaei S., Daryani S., Eslami-S Z., Samadi T., Jafarbeik-Iravani N., Bakhshayesh T.O., Majidzadeh-A K., Esmaeili R. (2018). Exosomes in Cancer Liquid Biopsy: A Focus on Breast Cancer. Mol. Ther. Nucleic Acids.

[8] Sandfeld-Paulsen B., Jakobsen K.R., Bæk R., Folkersen B.H., Rasmussen T.R., Meldgaard P., Varming K., Jørgensen M.M., Sorensen B.S. (2016). Exosomal Proteins as Diagnostic Biomarkers in Lung Cancer. J. Thorac. Oncol..

[9] Xu G., Jin J., Fu Z., Wang G., Lei X., Xu J., Wang J. (2025). Extracellular vesicle-based drug overview: Research landscape, quality control and nonclinical evaluation strategies. Signal Transduct. Target. Ther..

[10] Kalluri R., LeBleu V.S. (2020). The biology, function, and biomedical applications of exosomes. Science.

[11] He X., Park S., Chen Y., Lee H. (2021). Extracellular Vesicle-Associated miRNAs as a Biomarker for Lung Cancer in Liquid Biopsy. Front. Mol. Biosci..

[12] Xu R., Rai A., Chen M., Suwakulsiri W., Greening D.W., Simpson R.J. (2018). Extracellular vesicles in cancer—Implications for future improvements in cancer care. Nat. Rev. Clin. Oncol..

[13] Becker A., Thakur B.K., Weiss J.M., Kim H.S., Peinado H., Lyden D. (2016). Extracellular Vesicles in Cancer: Cell-to-Cell Mediators of Metastasis. Cancer Cell.

[14] Assarsson E., Lundberg M., Holmquist G., Björkesten J., Thorsen S.B., Ekman D., Eriksson A., Rennel Dickens E., Ohlsson S., Edfeldt G. (2014). Homogenous 96-Plex PEA Immunoassay Exhibiting High Sensitivity, Specificity, and Excellent Scalability. PLoS ONE.

[15] Ren A.H., Diamandis E.P., Kulasingam V. (2021). Uncovering the Depths of the Human Proteome: Antibody-based Technologies for Ultrasensitive Multiplexed Protein Detection and Quantification. Mol. Cell. Proteom..

[16] Nielsen J.E., Pedersen K.S., Vestergård K., Maltesen R.G., Christiansen G., Lundbye-Christensen S., Moos T., Kristensen S.R., Pedersen S. (2020). Novel Blood-Derived Extracellular Vesicle-Based Biomarkers in Alzheimer’s Disease Identified by Proximity Extension Assay. Biomedicines.

[17] Harris P.A., Taylor R., Thielke R., Payne J., Gonzalez N., Conde J.G. (2009). Research electronic data capture (REDCap)—A metadata-driven methodology and workflow process for providing translational research informatics support. J. Biomed. Inform..

[18] Harris P.A., Taylor R., Minor B.L., Elliott V., Fernandez M., O’Neal L., McLeod L., Delacqua G., Delacqua F., Kirby J. (2019). The REDCap consortium: Building an international community of software platform partners. J. Biomed. Inform..

[19] Krzyslak H., Szejniuk W.M., Falkmer U., Honoré B., Jørgensen M.M., Sten C., Pedersen S., Christiansen G., Kristensen S.R. (2026). Methodological and Short-Term Diurnal Variation in Surface and Cargo Proteins in Plasma Extracellular Vesicles. Curr. Issues Mol. Biol..

[20] Eisenhauer E.A., Therasse P., Bogaerts J., Schwartz L.H., Sargent D., Ford R., Dancey J., Arbuck S., Gwyther S., Mooney M. (2009). New response evaluation criteria in solid tumours: Revised RECIST guideline (version 1.1). Eur. J. Cancer.

[21] Thomas A., Mian I., Tlemsani C., Pongor L., Takahashi N., Maignan K., Snider J., Li G., Frampton G., Ali S. (2020). Clinical and Genomic Characteristics of Small Cell Lung Cancer in Never Smokers: Results from a Retrospective Multicenter Cohort Study. Chest.

[22] Barbone F., Bovenzi M., Cavallieri F., Stanta G. (1997). Cigarette Smoking and Histologic Type of Lung Cancer in Men. Chest.

[23] Lubin J.H., Couper D., Lutsey P.L., Woodward M., Yatsuya H., Huxley R.R. (2016). Risk of cardiovascular disease from cumulative cigarette use and the impact of smoking intensity. Epidemiology.

[24] Zou X., Zou S., Guo Y., Peng D., Min H., Zhang R., Qin R., Mai J., Wu Y., Sun X. (2023). Association of smoking status and nicotine dependence with multi-morbidity in China: A nationally representative crosssectional study. Tob. Induc. Dis..

[25] Théry C., Witwer K.W., Aikawa E., Alcaraz M.J., Anderson J.D., Andriantsitohaina R., Antoniou A., Arab T., Archer F., Atkin-Smith G.K. (2018). Minimal information for studies of extracellular vesicles 2018 (MISEV2018): A position statement of the International Society for Extracellular Vesicles and update of the MISEV2014 guidelines. J. Extracell. Vesicles.

[26] Johnsen K.B., Gudbergsson J.M., Andresen T.L., Simonsen J.B. (2019). What is the blood concentration of extracellular vesicles? Implications for the use of extracellular vesicles as blood-borne biomarkers of cancer. Biochim. Biophys. Acta (BBA)—Rev. Cancer.

[27] Simonsen J.B. (2017). What are we looking at? Extracellular vesicles, lipoproteins, or both?. Circ. Res..

[28] Nielsen J.E., Honoré B., Vestergård K., Maltesen R.G., Christiansen G., Bøge A.U., Kristensen S.R., Pedersen S. (2021). Shotgun-based proteomics of extracellular vesicles in Alzheimer’s disease reveals biomarkers involved in immunological and coagulation pathways. Sci. Rep..

[29] He X.Y., Zheng Y.X., Yang H.-S., Yu H.-Z., Xia Q.-J., Wang T.-H. (2024). PDGFBB facilitates tumorigenesis and malignancy of lung adenocarcinoma associated with PI3K-AKT/MAPK signaling. Sci. Rep..

[30] Donnem T., Al-Saad S., Al-Shibli K., Andersen S., Busund L.-T., Bremnes R.M. (2008). Prognostic impact of platelet-derived growth factors in non-small cell lung cancer tumor and stromal cells. J. Thorac. Oncol..

[31] Webber J., Steadman R., Mason M.D., Tabi Z., Clayton A. (2010). Cancer exosomes trigger fibroblast to myofibroblast differentiation. Cancer Res..

[32] Hong C.-S., Muller L., Whiteside T.L., Boyiadzis M. (2014). Plasma exosomes as markers of therapeutic response in patients with acute myeloid leukemia. Front. Immunol..

[33] Yu P., Han Y., Meng L., Tang Z., Jin Z., Zhang Z., Zhou Y., Luo J., Luo J., Han C. (2024). The incorporation of acetylated LAP-TGF-β1 proteins into exosomes promotes TNBC cell dissemination in lung micro-metastasis. Mol. Cancer.

[34] Hussen B.M., Saleem S.J., Abdullah S.R., Mohamadtahr S., Hidayat H.J., Rasul M.F., Taheri M., Kiani A. (2023). Current landscape of miRNAs and TGF-β signaling in lung cancer progression and therapeutic targets. Mol. Cell. Probes.

[35] Murai F., Koinuma D., Shinozaki-Ushiku A., Fukayama M., Miyaozono K., Ehata S. (2015). EZH2 promotes progression of small cell lung cancer by suppressing the TGF-β-Smad-ASCL1 pathway. Cell Discov..

[36] Chen G., Huang A.C., Zhang W., Zhang G., Wu M., Xu W., Yu Z., Yang J., Wang B., Sun H. (2018). Exosomal PD-L1 contributes to immunosuppression and is associated with anti-PD-1 response. Nature.

[37] Kim D.H., Kim H., Choi Y.J., Kim S.Y., Lee J.E., Sung K.J., Sung Y.H., Pack C.G., Jung M.K., Han B. (2019). Exosomal PD-L1 promotes tumor growth through immune escape in non-small cell lung cancer. Exp. Mol. Med..

[38] Shimada Y., Matsubayashi J., Kudo Y., Maehara S., Takeuchi S., Hagiwara M., Kakihana M., Ohira T., Nagao T., Ikeda N. (2021). Serum-derived exosomal PD-L1 expression to predict anti-PD-1 response and in patients with non-small cell lung cancer. Sci. Rep..

[39] Li C., Matsubayashi J., Kudo Y., Maehara S., Takeuchi S., Hagiwara M., Kakihana M., Ohira T., Nagao T., Ikeda N. (2019). Clinical significance of PD-L1 expression in serum-derived exosomes in NSCLC patients. J. Transl. Med..

[40] Wu K., Yu S., Liu Q., Bai X., Zheng X., Wu K. (2017). The clinical significance of CXCL5 in non-small cell lung cancer. OncoTargets Ther..

[41] Simoncello F., Piperno G.M., Caronni N., Amadio R., Cappelletto A., Canarutto G., Piazza S., Bicciato S., Benvenuti F. (2022). CXCL5-mediated accumulation of mature neutrophils in lung cancer tissues impairs the differentiation program of anticancer CD8 T cells and limits the efficacy of checkpoint inhibitors. OncoImmunology.

[42] Deng J., Jiang R., Meng E., Wu H. (2022). CXCL5: A coachman to drive cancer progression. Front. Oncol..

[43] Ullah A., Pulliam S., Karki N.R., Khan J., Jogezai S., Sultan S., Muhammad L., Khan M., Jamil N., Waheed A. (2022). PD-L1 Over-Expression Varies in Different Subtypes of Lung Cancer: Will This Affect Future Therapies?. Clin. Pract..

[44] Khan P., Fatima M., Khan A., Batra S.K., Nasser M.W. (2022). Emerging role of chemokines in small cell lung cancer: Road signs for metastasis, heterogeneity, and immune response. Semin. Cancer Biol..

[45] Geens M., Stappers S., Konings H., De Winter B.Y., Specenier P., Van Meerbeeck J.P., Verpooten G.A., Abrams S., Janssens A., Peeters M. (2021). Epidermal growth factor as a potential prognostic and predictive biomarker of response to platinum-based chemotherapy. PLoS ONE.

[46] Fujita S., Masago K., Hatachi Y., Fukuhara A., Hata A., Kaji R., Kim Y.H., Mio T., Mishima M., Katakami N. (2010). Genetic polymorphisms in the endothelial nitric oxide synthase gene correlate with overall survival in advanced non-small-cell lung cancer patients treated with platinum-based doublet chemotherapy. BMC Med. Genet..

[47] Liu C.-Y., Wang C.-H., Chen T.-C., Lin H.-C., Yu C.-T., Kuo H.-P. (1998). Increased level of exhaled nitric oxide and up-regulation of inducible nitric oxide synthase in patients with primary lung cancer. Br. J. Cancer.

[48] Colakogullari M., Ulukaya E., Yilmaztepe A., Ocakoglu G., Yilmaz M., Karadag M., Tokullugil A. (2006). Higher serum nitrate levels are associated with poor survival in lung cancer patients. Clin. Biochem..

[49] Chen G.G., Lee T.W., Xu H., Yip J.H.Y., Li M., Mok T.S.K., Yim A.P.C. (2008). Increased inducible nitric oxide synthase in lung carcinoma of smokers. Cancer.

[50] Masri F.A., Comhair S.A.A., Koeck T., Xu W., Janocha A., Ghosh S., Dweik R.A., Golish J., Kinter M., Stuehr D.J. (2005). Abnormalities in Nitric Oxide and Its Derivatives in Lung Cancer. Am. J. Respir. Crit. Care Med..

[51] Kaira K., Imai H., Mouri A., Hashimoto K., Miura Y., Shiono A., Yamaguchi O., Kobayashi K., Kawasaki T., Yasuda M. (2023). Clinicopathological impact of VEGFR2 and VEGF-C in patients with EGFR-major mutant NSCLC receiving osimertinib. Thorac. Cancer.

[52] Young R.J., Tin A.W., Brown N.J., Jitlal M., Lee S.M., Woll P.J. (2012). Analysis of circulating angiogenic biomarkers from patients in two phase III trials in lung cancer of chemotherapy alone or chemotherapy and thalidomide. Br. J. Cancer.

[53] Joo H.P., Park K.J., Kim Y.S., Sheen S.S., Lee K.S., Lee H.N., Oh Y.J., Hwang S.C. (2007). Serum Angiopoietin-2 as a Clinical Marker for Lung Cancer. Chest.

[54] Cañadas I., Taus Á., Villanueva X., Arpí O., Pijuan L., Rodríguez Y., Menéndez S., Mojal S., Rojo F., Albanell J. (2015). Angiopoietin-2 is a negative prognostic marker in small cell lung cancer. Lung Cancer.

[55] Daly S., Kubasiak J.C., Rinewalt D., Pithadia R., Basu S., Fhied C., Lobato G.C., Seder C.W., Hong E., Warren W.H. (2014). Circulating Angiogenesis Biomarkers Are Associated with Disease Progression in Lung Adenocarcinoma. Ann. Thorac. Surg..

