# Supplementary material for: Novel Blood-Based Extracellular Vesicle-Derived Biomarkers in Small Cell Lung Cancer Identified via Proximity Extension Assay
Source: Cancers (Basel). 2026 Feb 10;18(4):580. doi: 10.3390/cancers18040580 (PMC12938899; doi:10.3390/cancers18040580)
Supplement: Supplementary file 1 [file cancers-18-00580-s001.zip › Suplementary Tables S2 and S3.pdf]

Table S2. Summary of all significant proteins detected in the filtered dataset, with expression levels, raw and corrected *p*-values.

Mean protein NPX values Pt-BE vs HC						
**Protein**	**UniProt**	**Mean HC**	**Mean Pt-BE**	**Difference**	***p*-value**	**FDR**
PDGF subunit B	P01127	4.25	8.28	4.04	<0.0001	<0.0001
CXCL5	P42830	1.50	5.20	3.70	<0.0001	<0.0001
CCL17	Q92583	2.82	6.35	3.53	<0.0001	<0.0001
EGF	P01133	2.32	5.67	3.35	<0.0001	<0.0001
LAP TGF-beta-1	P01137	3.00	6.34	3.34	<0.0001	<0.0001
CD40-L	P29965	2.23	5.48	3.25	<0.0001	<0.0001
PD-L1	Q9NZQ7	2.77	5.78	3.01	<0.0001	<0.0001
VEGFA	P15692	1.82	4.56	2.74	<0.0001	<0.0001
ANGPT1	Q15389	1.70	4.38	2.68	<0.0001	<0.0001
CXCL1	P09341	3.71	6.36	2.65	<0.0001	<0.0001
CXCL11	O14625	4.01	6.07	2.06	<0.0001	<0.0001
TWEAK	O43508	0.66	2.72	2.06	<0.0001	<0.0001
MCP-4	Q99616	3.74	5.77	2.03	<0.0001	<0.0001
CD40	P25942	9.87	11.88	2.01	<0.0001	<0.0001
CASP-8	Q14790	1.98	3.83	1.84	<0.0001	<0.0001
IL18	Q14116	0.80	2.49	1.69	<0.0001	<0.0001
HGF	P14210	1.54	3.22	1.67	<0.0001	<0.0001
IL8	P10145	1.39	3.06	1.67	<0.0001	<0.0001
MCP-2	P80075	1.61	3.26	1.65	<0.0001	<0.0001
Gal-1	P09382	2.13	3.49	1.35	<0.0001	<0.0001
MMP12	P39900	0.58	1.83	1.25	<0.0001	<0.0001
TNFSF14	O43557	1.21	2.39	1.18	<0.0001	<0.0001

Mean protein NPX values Pt-BE vs HC						
**Protein**	**UniProt**	**Mean HC**	**Mean Pt-BE**	**Difference**	***p*-value**	**FDR**
CD244	Q9BZW8	5.14	6.29	1.15	<0.0001	<0.0001
ADA	P00813	3.22	4.28	1.06	<0.0001	<0.0001
ANGPT2	O15123	1.08	2.10	1.01	<0.001	<0.001
CCL4	P13236	0.82	1.80	0.98	<0.0001	<0.0001
CXCL13	O43927	4.42	5.31	0.89	0.007	0.011
IFN-gamma	P01579	1.96	2.82	0.86	<0.0001	<0.0001
MMP7	P09237	4.01	4.81	0.80	<0.001	0.001
TNFRSF4	P43489	2.67	3.37	0.71	<0.0001	<0.001
GZMA	P12544	3.92	4.52	0.60	0.029	0.046
CCL3	P10147	0.77	1.34	0.57	<0.001	<0.001
MCP-1	P13500	3.26	3.78	0.52	<0.001	<0.001
TNFRSF21	O75509	1.88	1.42	-0.47	<0.001	<0.001
CD5	P06127	7.20	6.64	-0.56	0.017	0.028
CD27	P26842	2.98	2.29	-0.69	<0.001	0.002

Table S3. Summary of all ROC curves for significant proteins between CN and BE, including AUC and predictive assessment and 95% CI.

**ROC summary Pt-BE vs HC (BH-significant proteins)**				
**Protein**	**AUC**	**95% CI**	**Sensitivity (%)**	**Specificity (%)**
PD-L1	0.99	0.96-1.00	93	96
CD40-L	0.98	0.95-1.00	93	96
LAP TGF-beta-1	0.98	0.94-1.00	100	96
PDGF subunit B	0.98	0.94-1.00	97	96
EGF	0.97	0.93-1.00	93	96
CASP-8	0.97	0.94-1.00	97	86
VEGFA	0.97	0.94-1.00	86	96
ANGPT1	0.97	0.92-1.00	97	96
CD40	0.97	0.92-1.00	93	96
CXCL1	0.96	0.92-1.00	93	93
CXCL11	0.96	0.92-1.00	83	100
TWEAK	0.96	0.91-1.00	97	93
CCL17	0.96	0.91-1.00	90	96
HGF	0.95	0.90-1.00	90	93
CXCL5	0.95	0.89-1.00	90	93
IL18	0.94	0.88-1.00	90	89
IL8	0.93	0.87-0.99	86	89
MCP-2	0.92	0.84-1.00	86	93
CCL4	0.91	0.84-0.98	93	79
TNFSF14	0.91	0.83-0.99	90	86
Gal-1	0.90	0.82-0.98	83	86
MCP-4	0.89	0.80-0.98	83	89
CD244	0.88	0.80-0.97	83	86

ROC summary Pt-BE vs HC (BH-significant proteins)				
**Protein**	**AUC**	**95% CI**	**Sensitivity (%)**	**Specificity (%)**
ADA	0.86	0.77-0.96	72	89
MMP7	0.83	0.73-0.94	62	93
IFN-gamma	0.83	0.72-0.94	90	71
ANGPT2	0.82	0.71-0.93	90	68
MMP12	0.82	0.69-0.94	69	100
CCL3	0.82	0.70-0.93	83	75
TNFRSF4	0.79	0.67-0.91	79	71
MCP-1	0.78	0.66-0.90	86	61
CXCL13	0.70	0.56-0.84	45	93
GZMA	0.65	0.51-0.79	31	100
CD5	0.33	0.18-0.47	24	79
CD27	0.28	0.14-0.42	100	0
TNFRSF21	0.19	0.07-0.30	3	100
